# Inclusivity in Practice Education: The Views and Experiences of Nurse Educators Supporting Nursing Students With Mental Health Needs in Mental Health Clinical Placements

**DOI:** 10.1002/nop2.70119

**Published:** 2025-01-31

**Authors:** Pras Ramluggun, Sarah Mansbridge, Rita Bhundoo‐Swift, Mahmood Anjoyeb, Margaret Rioga

**Affiliations:** ^1^ Northumbria University Newcastle ‐ Upon‐Tyne Northumbria United Kingdom; ^2^ Oxford Heath NHS Foundation Trust Littlemore Mental Health Centre Oxford UK; ^3^ Buckinghamshire New University London UK; ^4^ Institute of Health and Social Care Buckinghamshire New University Wycombe Buckinghamshire UK

**Keywords:** mental health nursing placement, mental wellbeing, nurse educators, nursing students, nursing students' mental health, nursing students' practice learning, practice assessors, practice supervisors, reasonable adjustment

## Abstract

**Aim:**

This study aimed to explore the perspectives and experiences of Nursing Practice Supervisors and Assessors, referred to as Nurse Educators, regarding their support for students with mental health needs during mental health clinical placements.

**Design:**

A qualitative survey design was employed to delve into Nurse Educators' views and experiences at two Mental Health Trusts in the Southeast of England, UK.

**Methods:**

Data were collected using Google Forms to create an anonymous online questionnaire. Thirty‐five Nurse Educators, comprising Practice Supervisors and Practice Assessors from two National Health Trusts, providing placements for two universities in the Southeast of England, responded to the survey. Thematic analysis was conducted to interpret the responses.

**Results:**

Factors which facilitated and inhibited Nurse Educators efforts to supporting students' mental health needs during placements were identified. They encompassed personal, professional, and procedural issues which were intricately intertwined to support students with mental health needs. While most Nurse Educators expressed a willingness to support students with mental health needs, challenges regarding the reasonableness of adjustments and their impact on Nurse Educators' practice were widely reported.

Recommendations include a review of learning arrangements, guidelines, and policies for students with mental health needs in clinical placements, as well as tailored training of students' inclusive needs for Nurse Educators.

No patient or public contribution.

## 1 Introduction

Preregistration nursing education varies across the world in terms of educational structure, curricular programme duration and content, due to the differences in healthcare systems, educational ideas and the regulatory bodies which set the standards for nursing education and approve the nursing programme. Hence, the amount and nature of the clinical placement and practice in the preregistration nursing programme can also vary.

In the UK the approved education institutes (AEIs), universities and other higher education institutes and practice learning partners have a collective responsibility for providing practice education that enables nursing students to meet their learning outcomes and supporting them with any related challenges to their learning in clinical placement. This includes providing students with a conducive practice learning environment equipped with dedicated practice learning staff and a faculty staff (commonly known as the Link Lecturer) linked to their clinical placement.

Under the changes for Nursing and Midwifery Council (NMC) standards for student supervision and assessment which came into effect in 2018 (updated in 2023), students are now assigned nominated Practice Supervisors and a Practice Assessor who support them to meet their respective preregistration nursing programme outcomes and the NMC standards in practice learning (Nursing and Midwifery Council 2023). It also includes an Academic Assessor (AA) who works with the nominated Practice Assessor to make recommendations for the student's progression for the assessment of their practice learning. Currently the roles of the AA and the former Link Lecturer (LL) coexist in some AEIs, but how they operate differs from each institution.

The Practice Supervisor can be any registered health and social care professional with the relevant up‐to‐date knowledge and experience for the preregistration nursing programme of the students they are supervising. The Practice Assessor is a registered nurse who has current knowledge and experience for the proficiencies and preregistration nursing programme outcomes of the students they are assessing (Nursing and Midwifery Council 2023).

Under the widening participation agenda aimed at addressing underrepresentation in accessing higher education, AEIs have a responsibility to provide inclusive and tailored learning experiences to the diverse needs of individual students (Office for Students 2023). This includes assisting Nursing Educators in implementing reasonable adjustments where appropriate, as identified in the student's inclusive support plan for students with mental health needs. These adjustments refer to modifications or accommodations to provide an equitable learning environment for students with disabilities to enable them to participate fully in their educational programme including clinical placements. For clinical placements these adjustments would require Nurse Educators to plan the practice learning and assessment with the student and the academic assessor to reasonably meet the student's specific needs without jeopardising the required professional standards of proficiency and values.

The impact of reasonable adjustments on students is meant to reduce barriers to learning in practice and enhanced their accessibility to practice learning. It is intended to help them to more effectively manage their health and wellbeing by reducing any risk of exacerbating their mental health conditions. By levelling the playing field, the reasonable adjustments are intended to enable students to perform to best of their abilities in achieving the proficiencies and programmes outcomes (Ramluggun, Jackson, and Usher 2021).

For Nurse educators the students' reasonable adjustments imply an increasing awareness and sensitivity to their diverse learning needs. In their professional development as nurse educators may require knowledge and skills in coordinating and collaborating with AEIs in supervising and assessing students practice learning.

However, the implementation of inclusive learning and assessment is fraught with difficulties for students with mental health needs (Ramluggun et al. 2018). Reasonable adjustments cannot always be easily implemented either in the classroom (Hughes and Byrom 2019) or in clinical placement to meet the NMC proficiency standard (Ramluggun 2020). The practice education of the preregistration nursing programme is recognised as being more challenging and stressful than the academic component (Onieva‐Zafra et al. 2020). Universities Uk (2023) recognises that better support is needed to reduce the mental health and wellbeing risks of students on placement. Understanding the views, experiences, and expectations of Nurse Educators are important for students' successful practice learning outcomes. However, the experiences of Nurse Educators in mental health settings are underexplored (Benny, Porter, and Joseph 2022). At the time of conducting this study, we found no other recent research specifically examining how Nurse Educators in the UK support students with mental health needs during placement.

## 2 Background

### 2.1 Clinical Learning Environment

In the clinical learning environment nursing students are supported to achieve their proficiencies by a range of healthcare professionals. Situated learning theorists such as Lave and Wenger (1991) to education argue that knowledge and learning are in experience. This view is supported by experiential learning theory (Kolb's 1984) advocating for a humanistic and constructivist approaches to education in transforming experience into knowledge. Hence, practice education is context dependent and influenced by authentic patient care situations and challenges. Applying situated learning principles to clinical practice, meaningful practical learning experiences can support the development of students' nursing knowledge, skills, clinical reasoning, and professional identity. Historically, nursing students were assigned a mentor in the United Kingdom (UK) to facilitate their learning and assess proficiencies in practice in a one‐to‐one relationship under the guidance of a nurse mentor helping students to adapt to their learning in the clinical environment. Communities of Practice theory developed by Lave and Wenger (1991) posit the notion of the social processes for legitimate peripheral participation (Lave 1991) where people with common interest for a subject area interact regularly, collaborate, and learn how to do things better. Hence, this includes instilling a sense of belonging in students to the clinical settings (Sherrod, Holland, and Battle 2020), so they feel part of the clinical team, which is important to promote effective clinical learning experience (Singer, Sapp, and Baker 2022). However, recognising the need for a more sustainable, creative, and flexible standard of student supervision and assessment, the prescriptive one‐to‐one mentoring approach which required students to spend 40% of their time with a dedicated mentor was discontinued (Leigh and Roberts 2018). Among the several reported challenges constraining the facilitation of practice learning mentorship responsibilities, prohibitive workload resulting in lack of adequate time to supervise students (Whaley, Hay, and Knight 2023) and managing the diverse students' learning needs (Ramluggun et al. 2022) featured prominently.

Clinical placement issues such as the inability to cope with the demands of the clinical environment are an important contributor to students' attrition (De Leon 2018) which impacts on the nursing workforce with nearly 47,000 nursing vacancies in the NHS at the end of 2022 (NHS Digital 2022). The level of stress experienced by students varies depending on the clinical experience during their preregistration nursing programme such as, when faced with the realities of their first clinical placement (Stones and Glazzard 2019), the application of knowledge and skills to different contexts of care and building relationship with clinical practice staff (Mazalová, Gurková, and Štureková 2022). Nursing students are often tasked with meeting multiple deadlines, balancing academic assignments with the attainment of clinical proficiencies during their clinical placements. These negative clinical experiences can impact on students' academic performance, mental well‐being (Brown et al. 2016) and may lead to their withdrawal from the nursing programme (Bakker et al. 2019). The relative of a nursing student, who tragically took his own life while on placement, emphasised the critical need for students to avoid feeling isolated during their time in clinical settings (Royal College of Nursing 2022). The sense of isolation, particularly during block placements lasting several weeks, poses a significant challenge for some students, who may feel disconnected from the support network available on campus. Accessing mental health support at university while on placement proves to be practically challenging for students due to limited‐service hours (Galvin et al. 2015; Ramluggun et al. 2018).

### 2.2 Students' Mental Health

Mental health is defined as “a state of mental well‐being that enables people to cope with the stresses of life, realise their abilities, learn well and work well, and contribute to their community” (World Health Organisation 2022) The mental health of students in higher education has been widely reported to be a global public concern (Ohadomere and Ogamba 2021) with reports that an equal number of students have experienced mental distress prior to their studies and developing symptoms during their studies (Grøtan, Sund, and Bjerkeset 2019). Higher education students' mental health is particularly at risk during their transition to adulthood (Cage et al. 2021) which is the onset of most mental health disorders (Solmi et al. 2021). At different stages of the student journey there are numerous challenges and stressors in managing university life such as academic, financial responsibilities and peer relationship impacting on student's mental health (Lattie, Stiles‐Shields, and Graham 2022) and this includes mature students as well (Lalatendu, Lan, and Collins 2018). The level and nature of stress varies in nursing students depending on the field of nursing. In the realm of healthcare services, stress is pervasive, often stemming from various factors such as interruptions during patient care, collaborating with inexperienced staff, resource shortages, and understaffing (Gopee 2018).

Furthermore, under the widening participation and the Equality Act (2010) which outlawed discrimination for applicants with protected characteristics seeking admission to higher education, more students from underrepresented groups are accessing and benefiting from higher education (Younger et al. 2019). As a result, there has been an increasing number of students with pre‐existing mental health issues enrolling on the preregistration mental health nursing programme (Ramluggun et al. 2018). These students can be at greater risk of experiencing mental health issues (Goodwill et al. 2018) as they face greater financial difficulties, with a lower feeling of belonging to university and find it more challenging to adapt to the norms and culture of university life and manage the demands of the preregistration nursing programme (Ramluggun et al. 2018). This trend was particularly pronounced in the wake of the Covid‐19 pandemic (Catling et al. 2022), which impacted on nursing students' practice learning. During this unparalleled period, students grappled with feelings of uncertainty and isolation (Henshall et al. 2023), compounded by fear and anxiety stemming from perceived Covid‐19 risks (Ramluggun 2020).

### 2.3 Mental Health Clinical Placement

Compared to other clinical nursing settings research indicates that mental health clinical placements represent one of the most formidable challenges in nursing education. These challenges vary depending on the educational pathway's requirement for experience in mental health practice. In some countries, mental health nursing is integrated into general nursing education, where students receive training in mental health care as part of their overall nursing curriculum. However, other countries such as the UK offer a distinct mental health nursing programme that focus specifically on the skills and knowledge required to work in mental health settings (Chatterton 2015).

In countries which offer non‐specialist mental health nursing programme students are offered at least one mental health placement within its clinical rotations (Demir and Ercan 2018). In these countries the difficulties associated with mental health placements stem from students' preconceived notions about patients, often viewing them as potentially dangerous and prone to violence, especially during their initial exposure to mental health settings (Shaygan et al. 2023). The paucity of research on mental health nursing students' experiences of their mental health clinical placement in the UK seem to suggest that the initial placement can be overwhelming for some students (Galvin et al. 2015).

### 2.4 Theoretical Framework

Lazarus and Folkman (1984) can be used to explain the transactional relationship of nursing students with the clinical learning environment in how they respond to the stressors of their placement when faced with the unknown and uncertainty. For example, during the pandemic the additional stresses faced by nursing students exceeded their ability to cope which adversely impacted on their wellbeing (Hamadi et al. 2021).

Nursing students are at high‐risk of experiencing stress and anxiety (Aloufi et al. 2021). In addition to the challenges faced by higher education students such as financial stress and time management **i**n general during transition and adjustment to university life, nursing students also navigate their professional journey on their programme to achieve the required proficiency in knowledge, skills, and attributes as a registered nurse while managing their academic workload (Hwang and Kim 2022). Clinical placement consisting of 2300 clinical hours as stipulated by the Nursing and Midwifery Council (Nursing and Midwifery Council 2023) which comprehensively prepares students in achieving their proficiencies for their nursing field of practice can be anxiety provoking, highly challenging and a significant source of stress (Onieva‐Zafra et al. 2020). Additionally, nursing students with preexisting mental health needs find the demands of their preregistration nursing programme challenging, which may adversely impact on their mental wellbeing and ability to successfully complete their studies (Cleary et al. 2012) particularly mental health nursing students (Ramluggun et al. 2018). Their clinical placement may present as a trigger or cause stress impacting on their mental wellbeing (Hughes and Byrom 2019). They require additional support with their studies which are managed in the form of an individual support plan which include reasonable adjustments to their teaching and learning (Rodger et al. 2015) unless there are concerns about their fitness to practise. However, a health condition on its own does not usually lead to fitness to practise concerns if it is effectively managed.

### 2.5 Support for Students' Mental Wellbeing in Placement

Students are required to make a declaration of health and character and disclose any health conditions as an entry requirement on the preregistration nursing programme to study to be a nurse (Nursing and Midwifery Council 2019b). Failure to disclose may lead to impaired fitness to practise if it is found that students' adverse health may have impacted on their ability to practise safely and effectively. However, students do not always disclose information about a disability in a timely manner for a realistic appraisal and accommodations of their learning needs to enable a prompt implementation of inclusive learning, teaching, and assessment. The fear of being stigmatised for mental health issues is a barrier to seeking support by nursing students as identified by Morton (2022). Consequently, students tend to disclose their mental health issues when their mental health has been impacted by their inability to cope with the demanding nature of their preregistration nursing programme (Ramluggun et al. 2018). Where students have reluctantly disclosed their mental health issues in their clinical placements, they may be overly protected, limiting their practice learning opportunities (Ramluggun et al. 2018).

With calls for ongoing transformative processes for inclusivity in higher education in addition to the recent changes in the organisation of practice education, Nursing Educators may face different challenges in managing students' mental health in placement. In this context a better understanding of Nursing Educators experience which is underexplored is imperative to address any support they may need.

## 3 Methods

### 3.1 Study Design

Due to the sensitivity of the topic, a self‐administered anonymous online qualitative questionnaire was used to survey a purposeful sample of Nursing Educators across two NHS Mental Health Trust in the Southeast of England. A qualitative survey design was used as it offers a useful flexible qualitative research tool to garner potentially rich data on a topic which has not been researched and is not well understood (Braun et al. 2022). Sample sizes for qualitative surveys are typically larger than those for traditional qualitative studies, often ranging from 20 to over 100 participants, depending on the study's scope and objectives (Braun et al. 2022). The qualitative questionnaire consisted of nine open‐ended questions aimed at exploring Nursing Educators' narratives on their views and experiences of responding and supporting students with mental health needs on placement in mental health settings. The questionnaire encompassed considerations regarding how attending to students' mental health requirements might have influenced their duties and obligations as practice educators. It delved into the level of readiness in addressing and handling students with mental health needs during their practicum, as well as identifying measures that could enhance their preparedness in facilitating these students' learning experiences. This includes exploring avenues for seeking support and identifying supplementary resources to aid in this process.

### 3.2 Conceptual Framework

The conceptual framework for designing the online anonymous questionnaire considered the key roles and responsibilities of Nursing Educators, the theoretical framework of stress in nursing students and related literature on nursing students mental health. The online questionnaire was adopted from a previous study on the topic with faculty staff (Ramluggun et al. 2022) following consultation with the Practice Learning Leads and a piloting of the questionnaire with a sample of Nursing Educators in the two Mental Health Trusts. The analysis of the questionnaire was guided by an interpretive framework on how to collect and analyse the researchable issues on this sensitive topic. It sought to understand Nursing Educators' viewpoints which are experientially based (Creswell and Poth 2017).

### 3.3 Context

Significant changes have occurred in the United Kingdom regarding the supervision and assessment standards for students enrolled in NMC‐approved programs aimed at achieving NMC proficiencies and program outcomes. Prior to 2018, students were primarily supervised and assessed by a single mentor. The 2018 standards (Nursing and Midwifery Council 2018) ushered in greater innovation in practice learning, aligning with the evolving skills and knowledge demanded of nurses today.

The transition from the previous model, characterised by one‐on‐one mentorship where a single mentor bore most of the responsibility for support, education, and assessment (Nursing and Midwifery Council 2010), was deemed unsustainable and lacking in rigour. In contrast, the 2018 NMC standards underscore the interprofessional nature of practice, wherein all registered professionals can contribute to the learning process. This shift moves away from isolated training (Willis Commission 2012) towards fostering team‐based learning, thereby enhancing the validity and reliability of assessment through feedback sourced from a wider range of professionals.

Consequently, impactful changes to mentor roles were introduced, designating roles such as Practice Supervisors (PS), Practice Assessors (PA), and Academic Assessors (AA) to distribute responsibilities for supervision and assessment among multidisciplinary professionals from diverse backgrounds.

### 3.4 Data Collection

A Google Form was used for the online questionnaire to enable full anonymisation of participants. The questionnaire consisted of three parts. The first part included an introduction of the study and a mandatory consent form which needed to be completed to proceed to the next part which required participants to identify their professional backgrounds and their roles as practice supervisors/assessors. The final part comprised of nine open‐ended questions on participants' views and experiences in supporting and facilitating the practice learning of students with mental health needs (see Appendix A).

The study poster was promoted by gatekeepers, namely the NHS Hospital Trusts' Practice Learning Environment Lead/Practice Learning Facilitators, through email dissemination to the respective managers of the clinical placements. Data collection occurred between 26th September 2022 and 20th January 2023. Invitations containing participant information sheets and a survey link were distributed via email by the Practice Learning Environment Lead/Practice Facilitator from the two Mental Health Trusts.

Thirty‐five Nursing Educators, comprising Practice Supervisors and Assessors, responded and completed the questionnaire. The majority were Mental Health Nurses, with a few participants holding dual registrations in Mental Health and Adult Nursing, along with a small number from Adult and Children nurses who also work for the two Mental Health Trusts completed the survey. The tables below provide the participants' professional backgrounds, field of practice and their professional roles as nurse educators (Tables 1 and 2). Nurses with dual registration bring a unique and comprehensive skill set to Mental Health Trusts, enabling them to deliver high‐quality, holistic, evidence‐based, person‐centred care in a variety of settings. Their roles can vary widely, but generally include comprehensive physical and mental health assessments to understand the full scope of a patient's needs. They contribute to the development and implementation of integrated care plans that address complex coexisting physical and mental health conditions, ensuring a coordinated approach to patient care. They are instrumental in bridging the gap between physical and mental health care, providing a seamless, integrated service that enhances patient outcomes.

### 3.5 Participants Professional Backgrounds

**TABLE 1:** Participants' professional backgrounds and nursing field of practice.

Professional field of practice	Number of participants
Mental health nursing	30
Adult nursing	5
Children nursing	1
Learning disability	1

*Note:* A few participants had dual registrations.

**TABLE 2:** Participants' professional roles as nurse educators.

Role	Number of participants
Practice supervisors	11
Practice assessors	24

*Note:* It is important to note that by default Practice Assessors can also supervise students but cannot supervise students for whom they are nominated as Practice Assessors (see Appendix B for detailed information on participants ‘roles and professional backgrounds).

### 3.6 Ethical Considerations

The study was approved by the University's Research Ethics Committee (UREC Ref: Redacted). The invitation to participate in the study was in accordance with the Declaration of Helsinki. Participants' consent was sought and recorded on the questionnaire before accessing the questions. The participant information sheet detailed the purpose of the study including a link to the survey, the need for consent, and the assurance of anonymity. All data were collected anonymously and confidentially, and limited demographic information were collected to avoid identification of participants.

### 3.7 Data Analysis

Data was analysed using Braun and Clarke (2006) steps for thematic analysis to explore participants' views and experiences of supporting students with mental health needs. This method of data analysis is recommended when little is known about the topic and involves a systematic process of coding and identifying themes by interpreting experiences and perceptions in a systematic manner (Maguire and Delahunt 2017). The iterative process of the data analysis consisted of six steps starting with reading the participants' narratives repeatedly to become familiar with the data to derive codes which were close to the terms used by participants. The codes were then sorted based on how they were linked and related to develop meaningful categories for generating the themes (see Appendix C). To ensure inter‐coder reliability the systematic steps which included Independent coding of the data independently by the first author and the research assistant. Coding discussion meeting with the research assistant to discuss any challenging or ambiguous pieces of data and coding choices to work towards a consensus on how to apply codes in problematic areas, work towards a consensus on how to apply codes in problematic areas to build a shared understanding of the emerging themes. The themes were defined and labelled following discussion with the research team during joint coding sessions. The later process entailed revisiting the coded data to determine if the data sufficiently support the theme by checking if there was too much variation across text segments to justify the theme. This entail refining the themes which were too broad and abstract and finally agreeing the final themes and sub‐themes and locating examples to use as illustrative quotes.

## 4 Results

**FIGURE 1:**
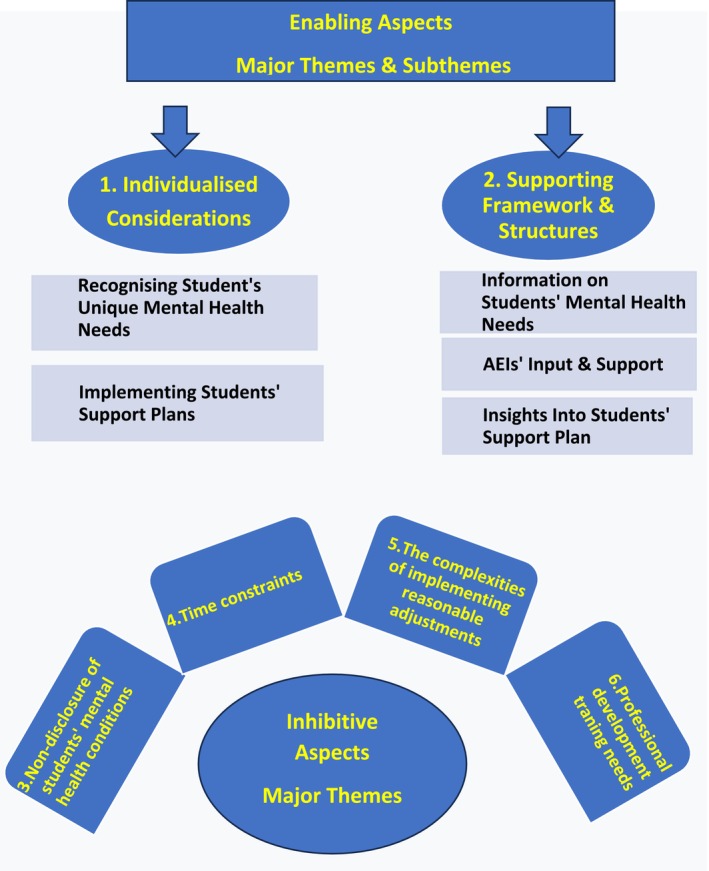
Overview of themes and subthemes.

Data analysis revealed two major themes that were significant in helping Nurse Educators to support students with mental health needs during placements. Additionally, four major themes were identified that hindered Nurse Educators' efforts to provide support, along with strategies to overcome these barriers. Figure 1 below provides an overview of the themes and subthemes, illustrating the enabling and inhibitive aspects of supporting students with mental health needs.

### 4.1 Major Theme 1: Individualised Considerations

#### 4.1.1 Subtheme 1.1: Recognising Students' Unique Mental Health Needs

Participants recognised the significance of being supportive by acknowledging and comprehending students' unique mental health needs. They emphasised the importance of addressing these individualised needs in assisting students with their practice learning requirements. Overall, participants with backgrounds in mental health nursing demonstrated greater understanding and empathy towards students with mental health needs.

Being understanding and supportive to help them meet their learning objectives. Each student is different; they must all be tailored to their individual needs.

[…] as mental health nurses it would come naturally to us to support students' mental health needs.

Seek their views on what support they may feel would be necessary.

#### 4.1.2 Subtheme 1.2: Insights Into Students' Support Plans

Participants took various actions to support students, such as allocating more time for them and confidentially offering a safe, non‐judgmental space. They actively sought the students' input on how best to support them, valuing their perspectives and preferences in the process.

Providing them more time […] monitor for any signs that the students are struggling.

Provide the student a safe and non‐judgemental space to express his/her mental health needs.

[…] we would in the first instance ask them how they can be supported.

### 4.2 Major Theme 2. Supporting Framework and Structures

#### 4.2.1 Subtheme 2.1: Information on Students' Mental Health Needs

Participants emphasised the necessity for greater clarity and transparency regarding students' support plans before the commencement of their placements. They expressed that such transparency would better prepare them for their roles. At times, they relied on students to disclose the support they required. However not all students are willing to or prepared to share information related to the support they may require.

Helpful to know if a student has experienced any particular trauma—bereavement etc. which may trigger mental health issues during the course of placement with us.

To be informed in advance so that we can make reasonable adjustments, including allocating an assessor who is equipped to support them.

The student being honest and open. This would allow staff to be more prepared and considerate of the needs of the student.

The student being defensive and not willing to open up or disclose their issues or need.

#### 4.2.2 Subtheme 2.2: AEIs' Input and Support

The participants emphasised the pivotal role of the Link Lecturer (LL) in ensuring that students receive the necessary support to achieve their learning objectives. They stressed the significance of cultivating a strong relationship with the LL and discussed how they would actively engage the LL to provide support for students with mental health needs.

Having a good link with the university link lecturer. I would refer to the link lecturer.

I had to involve the link lecturer to have a more comprehensive plan for the student to achieve their learning objectives.

I would seek support from the student's Link Lecturer and support groups if I am aware of recommended support services

Participants also emphasised the importance of providing information on university support services and guidance at the earliest opportunity on how to access them. This would ensure that students are effectively directed to the relevant support resources when needed.

Early sign posting of available recommended University Support Services

A list of different contacts for Health & Wellbeing would be helpful as well as university support services.

Assessors and supervisors should be informed on how to access support. Also, what are the support options?

#### 4.2.3 Subtheme 2.3: Insights Into Students' Support Plan

Participants who were familiar with the students' support plans found them beneficial. They shared how they effectively implemented these plans, providing examples of supportive adjustments to the students' practice learning. This included offering flexible working hours, reallocating specific tasks, all while ensuring that the learning objectives were met.

[…] it can help you to support the students the best way you can since it is individualised.

To look at the allocated task, not to put pressure and stress on the students.

Being flexible with the working conditions, taking into consideration their learning outcomes.

Conversely, participants also alluded to inhibiting factors in supporting students' mental health needs within clinical practice, which will be explored in greater depth in the following theme.

### 4.3 Major Theme 3: Non‐Disclosure of Students' Mental Health Conditions

Many participants felt constrained by the absence of clear guidance and not being alerted in a timely way on how to support students with mental health needs and placement expectations for students' reasonable adjustments. Owing to the sensitive nature of this information, students often waited until they were well into their placements before disclosing their support requirements to senior staff, who then relayed this information to the Nursing Educators.

University also don't alert the placement supervisors early enough of the student's mental health need and support in place from the university or reasonable adjustment expected from the placement.

In my experience students only disclose their mental health difficulties to senior staff on the ward because it is a sensitive and private matter. The ward manager had to speak to the nursing team which was much later into the student's placement.

Participants highlighted the difficulties of handling sensitive information about the student's mental health needs which they felt their colleagues may have overlooked. They expressed strong feelings about the potential consequences of mishandling such information and the impact it may have on the student. They also reported the challenges of raising concerns about the students' performance related to their mental health needs to university and the potential pressure to pass the students if it was their final placement.

Yes, especially if you are the first to escalate the mental health issue noticed which other staff tend to ignore. You then become the student and colleagues' enemy. It could be demoralising and humiliating if not handled carefully. Especially if the student is in the last placement and there is pressure from university, colleagues and student to pass the placement

Ultimately us raising concerns with the university regarding their performance on placement is difficult unless we have permission from them.

Participants also underlined how the lack of disclosure regarding mental health issues can undermine the student's confidence and cast doubts on their motivation to learn as they strive to achieve their proficiencies. They emphasised how not being aware of the students' needs can impact on their performance and the support they receive in placements.

The student appeared to struggle with staff and service users which made it seem as if he was not interested.

I think […] it really does affect the confidence of students who have worked so hard to build themselves up.

The university flagging at the onset of placement mental and physical health issues that could impact on the student's learning and performance and support required.

### 4.4 Major Theme 4: Time Constraints

Participants noted that the time available to support students fell short of their ideal standards. They highlighted those supporting students with mental health issues added significant demands to their workload, making it challenging to accommodate the students' additional learning needs.

Added demand is placed on the time that is not even sufficient to manage huge caseloads.

Need more time to accommodate their extra needs.

At times, this led to a notable delay in recognising students who were struggling due to their mental health issues. Consequently, participants experienced feelings of guilt for not providing adequate support to the students and felt that they were letting them down.

We need time, quality time to give to them otherwise we will fail them and ourselves.

I did find this whole experience daunting and guilty feelings that I have failed this student.

### 4.5 Major Theme 5 the Complexities of Implementing Reasonable Adjustments

Conversely, some participants noted that there were limitations to the accommodations that could be provided for students within clinical practice to facilitate their practice learning. These allowances included flexible working arrangements such as adjusted working hours and shift patterns, shorter and more frequent breaks, and access to office equipment like coloured paper and screens to aid in reading clinical notes and other medical documentation as needed. However, participants highlighted that making certain reasonable adjustments placed additional pressure on the rest of the nursing staff or team.

Additionally, participants underlined that students still need exposure and practical experience in meeting patients' needs, regardless of any reasonable adjustments made. They questioned whether these adjustments adequately reflected how the student would address the patients' needs effectively.

Perhaps what is missing from these debates is that patients' needs don't decrease in line with adjustments having to be made for the mental health of nursing professionals.

Only in as far as there is a greater emphasis on the mental health of the workforce now and with it a need for us to make reasonable adjustments for those involved, that in turn places a pressure on those left.

[…] only in the amount of sickness time they needed to take making it more difficult to achieve practice hours

They emphasised that a nurse's role is demanding both physically and mentally, underscoring the necessity for a resilient workforce capable of managing their own mental health and supporting others'. They questioned whether alternative career pathways should be considered for these students.

We need a workforce that comes with an ability and skill set to manage theirs and others mental health.

Undoubtedly, a nurse's job is hard physically/mentally and to do the job requires robustness in both areas. I have had occasion when I've had to discuss with staff nurse's non client facing roles as a career path for them, as they are not able to consistently carry out the client facing role.

They raised questions about the selection and recruitment process for admitting students into the program, as well as the necessity of reviewing students with mental health needs before they commence their placements. They expressed uncertainty regarding whether these students would be capable of fulfilling the responsibilities of a mental health nurse upon completing the program.

I think we need to go right back to how student nurses are selected, how aware are they of what the lived experience of being a nurse is and do they feel they can portray that role.

Perhaps being able to vet student nurses before they come on placement so we can pre‐empt any problems or, in some cases, advise against them coming to a particular placement.

Furthermore, they expressed unease about challenging students' practice to optimise their learning experience. They feared that they might be perceived as unsupportive of the students' mental health needs if they chose to intervene.

I think it might affect my work, since I might not feel comfortable to put too much pressure on them and challenge their practice, in case I am perceived as being judgemental towards their mental health.

From a practical perspective, participants also stated that absence, sickness, and lateness made it challenging for students with mental health to achieve the required practice hours:

Challenges are improvised absence or lateness to work or capacity to work or to take high load.

Only in the amount of sickness time they needed to take, making it more difficult to achieve practice hours.

It is notable that most participants reported having either no experience or very limited experience in implementing support plans for students with mental health issues. They suggested that raising awareness of the practice learning needs of students with mental health requirements, along with their support plans, and incorporating relevant assistance into assessor/supervisor training, would be beneficial.

[…] very useful to know learning styles and extra support we can offer.

I am not familiar with ISP; it would be useful to include this in the practice supervisor/assessor training.

Raise awareness of students with mental health difficulties as a potential tool to support them.

This underscores the significance of sharing any support plans with the placement team to guarantee an optimal clinical learning environment for students struggling with mental health challenges.

### 4.6 Major Theme 6: Professional Development Training Needs

Training emerged as a prevalent theme among participants as a continuous professional development need to promote inclusive clinical learning environment with a focus on enhancing understanding and support for students with mental health needs. The findings included the importance of being well‐informed about the specific support required for students, strategies for implementation, and avenues for additional support referral. Notably, individuals from non‐mental health backgrounds expressed interest in Mental Health First Aid training, aiming to better recognise signs and symptoms indicative of students experiencing mental health challenges. The training and support for Nurse Educators should also include how to balance students' support with academic integrity.

A list of different contacts for Health & Wellbeing would be helpful as well as university support services.

Mental Health first aid course should be offered to any registered nurse working with students.

Have some training around what signs & symptoms to look for if the student couldn't share their mental health condition with you, looking for clues.

I think the challenge is that there is only so much give/allowance one can make in a nurse's role before their effectiveness is nullified.

## 5 Discussion

Our findings indicate that there are personal, professional, and processual implications supporting nursing students with mental health needs in clinical placement and confirm similar findings for the theoretical component of the preregistration nursing programme (Ramluggun et al. 2022; Hughes and Byrom 2019). Overall, the findings indicated that Nursing Educators were willing to support the students to develop their skills and proficiencies, such as offering them flexible working hours and reviewing their allocated tasks in placement. There were some acknowledgments of useful structures and guidance for collaborative working with the university to support the students. However, they also presented some inherent challenges for students who require additional learning support arrangements.

The Link Lecturer was identified as being the most influential faculty staff in supporting the Nursing Educators within the learning environment as identified by other findings as a pivotal role to promoting communication between students, universities, and their placement (King 2018) and supporting them in placement (Stuhlmiller and Tolchard 2019). The Academic Assessor which is one of the three key roles in addition to the Practice Supervisor, Practice Assessor for supporting students' practice learning for the changes in the Nursing and Midwifery Council standards (Nursing and Midwifery Council 2023) was not mentioned. This is due to the specific arrangements at the universities in this study.

The findings from this study indicated that students with mental health needs can add to the Nurse Educators' workload, which is recognised as a demanding role (Benny, Porter, and Joseph 2022) because of the reported additional support for students in addition to their assigned workload. Nurse Educators felt constrained in their role to supporting students because they had to manage the limited time allocated for students, which was not always protected with their other caring responsibilities to patients. The inadequate protected time remains a prevailing issue despite the departure from the one‐to‐one mentoring approach for practice learning and is consistent with the only study so far (Whaley, Hay, and Knight 2023) which examined the changes to supporting and assessing students for the Nursing and Midwifery Council (2018) standards. Protected supervision time has been reported as being essential in building a positive relationship with students in clinical practice which can influence the students' sense of belonging, socialisation into the profession and overall engagement with learning (Jack et al. 2018). Positive relationships can empower students' autonomous learning (Partington, Brook, and McKeown 2024) and may also encourage open discussions about their individual learning needs in practice settings.

Hence, a review of Nurse Educators' protected time for allocated students with mental health needs in practice is worth considering.

Being unaware of students' mental health needs and their learning support arrangements, were viewed as a major barrier to supporting students' learning in placement. Feelings of frustration and guilt for being unable to adequately support students' practice learning in the absence of important information about students' mental health were reported in our findings. To meet the legal imperatives under the Equality Act (2010) the university policy and guidance on reasonable adjustments stipulates that where students have disclosed their health conditions, but do not wish for this information to be shared with practice learning partners to comply with data protection (Data Protection Act 2018) regulations their confidentiality can be protected. In such cases the Nursing and Midwifery Council policy on reasonable adjustments stipulates that the nature of the medical conditions would remain confidential by focusing and only sharing the reasonable adjustment arrangements for accessible learning in practice (Nursing and Midwifery Council 2019a). Still, the findings of our study indicate that Nursing Educators were either not always aware of students' reasonable adjustments before they start their placement or not always very clear on how to implement these arrangements when they became aware. It was not clear if this could be attributed to communication between the university and their practice learning partners, or it could be due to students not disclosing their mental health needs to the university. Students' health condition rarely prevents them from completing the course when they have sought help (Morton 2022) and implementation of reasonable adjustments can enhance the learning process and support students to develop their full potential (King 2018). However, the lack of disclosure in our findings is consistent with other studies which indicated students with a diagnosed condition may be managing their placement effectively until they incur an incident which exerts an excessive amount of stress and/or triggers a past trauma which inevitably adversely impacts their mental health (Moxham et al. 2024; Ramluggun et al. 2018; Cleary et al. 2012). The findings by Moxham et al. (2024) suggest that certain mental health clinical learning environments, such as non‐acute community placements, can foster more positive attitudes towards mental health, which may encourage students to seek support for their own mental health issues. However, since our study did not collect specific information on participants' placement settings, we cannot determine whether participants' responses were influenced by their clinical environments.

Although the Nursing and Midwifery Council discussed issues of confidentiality and supporting students with disabilities, there is limited advice given on how to encourage students to disclose or guidance for all concerned on their roles and responsibilities for students with mental health issues compared to other healthcare programmes. For example, the General Medical Council guidance on supporting disabled learners in medical education and training (General Medical Council 2019) and supporting students with mental health conditions (General Medical Council 2021). The Royal College of Nursing (Royal College of Nursing 2017) guide on reasonable adjustments provides some clarity on adjustments in the workplace effectively and emphasises the need for employees to be upfront about any impairments so employees are capable of safe and effective practice. The Royal College of Nursing (2024) Peer Support Service—Information for Students provides case studies illustrating how to support students with mental health issues and other disabilities in creating inclusive healthcare placements.

There are a range of factors that will influence disclosure for students, such as past negative experiences of disclosure, self‐stigma, lack of support, low self‐esteem, fear of the unknown (Cage et al. 2020). Stigma and stereotyping are a common barrier for disclosing mental health conditions (Kaiser et al. 2023) and this despite the raised awareness for good mental health and wellbeing. Debatably, disclosure in clinical practice can trigger professional conflicts for Nurse Educators in their roles as gatekeepers of their profession as identified in our findings. One of the key issues in our findings was Nurse Educators' views of the incompatibility of the extent of adjustments students were being afforded in practice to meet their proficiencies, its impact on their practice learning, assessment of proficiencies and how such adjustments would be incorporated upon registration as a nurse. The concept of reasonable adjustment is complex with varied interpretation and its implementation poses numerous challenges in preregistration education (Craig, Wakefield, and Pryjmachuk 2023). Depending on the nature of the adjustments this can limit the areas of work undertaken by the student or influence safeguarding thresholds (Demir and Ercan 2018). The adjustments must work for the employee and the employer as both sides must agree to the changes (Royal College of Nursing 2017). Nursing Educators raised ethical concerns about reasonableness of the adjustments which are not routinely made for other students which reduces their supervision time for these students and a level playing field for all students in providing equitable practice learning experience for all students. Notably, similar findings were found about these issues for faculty staff teaching on health and social care programmes (Ramluggun et al. 2022).

The ethical considerations raised by Nurse educators in seeking students' consent and maintaining the confidentiality for the information on students' mental health needs bears some similarity to the findings of another study on disclosure of such information in placement (Ramluggun et al. 2018). The findings from this study underscore the importance of fostering a supportive environment where students feel comfortable voluntarily disclosing their mental health issues. Providing clear information about the benefits and implications of such disclosures is crucial for implementing reasonable adjustments. This approach ensures that disclosure leads not to stigmatisation or discrimination but to the necessary support and adjustments that enhance students' wellbeing.

The benefits of people with lived experience such as peer support specialists in mental health care is well recognised, but how lived experiences of nursing students are managed on their programme is under examined (Ramluggun et al. 2018). In our findings Nursing Educators raised professional integrity concerns about the preregistration nursing programme and the nursing profession for the adjustments to enable students with mental health needs to meet the NMC standards. This raises areas of concerns such as students may fail to practise to their full potential if Nursing Educators are not aware of their mental health issues and the related support. The assessment of their practice learning may also be substandard if Nursing Educators are reluctant to grade students, accordingly, escalate concerns about their progress or even fail students with mental health needs who are not performing to the required standards because of the fear of being perceived as being discriminatory. The lack of consistency of assessment of students' competency to practice has been reported as a matter of concern (Helminen et al. 2017). Students' disability which includes mental health can be a factor for the failure to fail students (Neal‐Boylan, Michelle, and Lussier‐Duynstee 2021) and may influence Nurse Educators' assessment of their practice learning assessment. Therefore, it is important that Nurse Educators are adequately prepared and supported to supervise and assess students with mental health needs in placements.

Our findings revealed that most Nurse Educators expressed a need for training on how to effectively support students with mental health needs and promote an inclusive clinical learning environment. This includes tailoring professional development opportunities and activities on recognising signs and symptoms, especially for Nurse Educators from non‐mental health backgrounds. The primary focus of training was on implementing students' support plans, including necessary adjustments, and effectively signposting them to available support services. Interestingly, participants requested better information on where to refer students for university support but overlooked existing resources for student wellbeing within their own organisations (Mental Health Trusts). This underscores the importance of enhancing Nursing Educators' awareness of student support resources during practice supervisor/assessor training, ensuring they are equipped to recognise, respond to, and appropriately refer students with mental health needs to all accessible support services.

To effectively implement reasonable adjustments and support for students with mental health needs in clinical placements, there is a need to review the perception of reasonable adjustments in the context of competence and fitness to practice (Storr, Wray, and Draper 2011) as this is the main challenge for nurse educators and AEIs. Reasonable adjustments offer a plan on how nurse educators can support students to access their practice learning and this should be promoted by creating an inclusive clinical learning environment where learning and assessment strategies can be delivered flexibly to meet the needs of all students (Crawford et al. 2022). Nurse educators should have comprehensive support and training as part of the continuous professional development which could include; a better understanding of reasonable adjustments through scenario‐based workshops, promoting honest conversations on the challenges and practical solutions to implementing reasonable adjustments in practice and a better understanding of the statutory requirement for the students with disability mainly the Equality Act (2010) and discrimination Act of 2005, (Tee and Cowen 2012). Collaboration and communication are essential and the tripartite relationship between the Nurse Educators, Link lecturers, and students supports shared responsibility and partnership in implementing reasonable adjustments effectively within the clinical environment (Griffiths et al. 2010). This could include promoting Nurse Educators' self‐evaluation and reflection of their practice to enhance their professional development.

### 5.1 Implications for Practice Education

The clinical learning environment plays a vital role in nursing education, influencing students' growth, professional development, and the satisfaction they derive from their practice learning, which in turn impacts their retention in the preregistration nursing programme (Cant, Ryan, and Cooper 2021). The quality of the preregistration nursing programme hinges on the level of supervision and the robustness of assessment in the clinical placement (Immonen et al. 2019). Therefore, it is imperative for Academic Education Institutions (AEIs) and their practice learning partners to ensure that students of all backgrounds receive high‐quality clinical learning environments.

This may necessitate more active and sustained involvement of faculty staff in their roles as Academic Assessors and/or Link Lecturers. For instance, arranging a joint meeting with the student and their Nurse Educators at the onset of the placement allows for early identification of concerns and the implementation of support plans. Ongoing support during the placements will offer the students, Nurse Educators, and link lecturers the opportunity to proactively address any concerns and ensure the students' learning needs are being met effectively. However, there has been a noticeable decrease in the physical presence of faculty staff in placements following the pandemic, impacting their contribution to Practice Supervisor/Assessor training and updates in line with the current NMC standards for student supervision and assessment. Furthermore, faculty staff (Link Lecturers and/or Academic Assessors) sustained involvement in students' practice learning across AEIs varies Therefore, there is a clear need for AEIs to take a more active role with their practice partners in preparing Nursing Educators to provide inclusive support to students during placement as part of their continuous professional development.

Considering the potential benefits, widespread adoption of an adjustment passport, akin to the Access to Work Passport piloted by the Department of Work and Pensions (Department of Work and Pension 2021), could be worthwhile. This document, trialled for students with disabilities and complex needs at universities during the pandemic, could streamline information about the student's condition and necessary adjustments, eliminating the need for repetitive disclosures and facilitating earlier access to support for practice learning.

Furthermore, in addition to addressing the training needs of Nurse Educators, it is essential to evaluate how nursing students are prepared for the practice supervisor role during their formative years of the programme. Developing their awareness and relational skills to recognise and meet the diverse learning needs of the students they will supervise warrants careful review.

### 5.2 Strengths and Limitations

This qualitative study represents a pioneering exploration into Nurse Educators' experiences of supporting students with mental health needs in practice learning. It offers valuable empirical insights for promoting inclusive practice education and serves as a foundation for further investigations in diverse clinical learning environments.

While qualitative research aims for depth rather than generalizability, it is important to note that this study's findings are based on a purposive sample within a specific nursing practice field, limited to registered nurses. Thus, its applicability to other practice learning contexts and clinical settings may vary due to potential differences in student support arrangements.

Some participants' responses were constrained by shorter narratives, limiting the depth of detail provided. A deductive study with a larger sample examining surveying the trends and frequencies of the responses of Nurse Educators' views and experiences across various clinical settings, supplemented by in‐depth interviews from a representative sample, could offer a more nuanced exploration of the issues raised in this study.

Furthermore, future research endeavours should also focus on exploring the experiences and expectations of students with mental health needs regarding their practice learning. This holistic approach would contribute to a comprehensive understanding of the dynamics involved in supporting students with mental health needs in clinical settings.

## 6 Conclusion

This study represents the initial exploration into the experiences of Nurse Educators in facilitating the practice learning of students with mental health needs. It reveals a nuanced perspective regarding the endeavour to foster inclusive practice learning, which inevitably encounters challenges in managing students' mental health during placement. The findings underscore the necessity for faculty and their practice partners to acknowledge the complexities inherent in facilitating the practice learning of students with mental health needs. Supportive approaches, structures, and processes aimed at enhancing students' inclusive practice learning were identified. Further research is recommended to delve into the practice learning experiences of students with mental health needs.

## Author Contributions

P.R.: Conceptualization, design, data curation, analysis and interpretation, investigation, methodology, project administration, validation, visualisation, writing – critical review and editing. S.M.: Data curation, analysis and interpretation of data, methodology, project administration, validation, visualisation, writing – critical review and editing. R.B‐S.: Conceptualization, analysis and interpretation of data, visualisation, writing – critical review and editing. M.A.: Analysis and interpretation of data, validation, writing – critical review and editing. M.R.: Analysis and interpretation of data, validation, writing – critical review and editing.

## Conflicts of Interest

The authors declare no conflicts of interest.

## Data Availability

The data that support the findings of this study are available on request from the corresponding author. The data are not publicly available due to privacy or ethical restrictions.

## Appendix A Survey Questions

1. While supporting students' learning you may come across students with mental health needs. How do you think you can support these students?
2. Have you ever had any concerns for a student's mental health in placement? If yes, can you please tell us about this experience?
3. Who would you seek support from if you were concerned about a student's mental health?
4. Some students may have an Individual Support Plan (ISP) which identifies reasonable adjustments for their teaching and learning. What is your experience of implementing ISPs for students with mental health needs in placement?
5. If you have experienced any challenges in supporting students with mental health needs, what would you say was most challenging and why?
6. Has managing students' mental health needs in placement impacted on your work, and/or your health and wellbeing? If yes, in what way?
7. If you have answered yes to the previous question. Please tell us if you have accessed any support from the Trust's Health and Wellbeing Services?
8. What do you think would better prepare you in your role to work with students with mental health needs?
9. Do you have any other comments about working with students with mental health needs?

## Appendix B Participants' Roles and Professional Backgrounds

**Table:**

Participant number	Practice supervisor	Practice assessor	Mental health nursing	Adult nursing	Children nursing	Learning disability nursing
Participant 1	x	x	x	x		
Participant 2	x	x			x	
Participant 3	x		x			
Participant 4	x		x			
Participant 5	x			x		
Participant 6	x	x	x			x
Participant 7	x	x	x			
Participant 8	x	x	x			
Participant 9	x	x		x		
Participant 10		x		x		
Participant 11		x	x			
Participant 12	x	x	x			
Participant 13	x	x	x			
Participant 14	x	x	x			
Participant 15	x	x		x		
Participant 16	x		x			
Participant 17	x		x			
Participant 18	x	x	x			
Participant 19	x	x	x			
Participant 20	x	x	x			
Participant 21	x	x	x			
Participant 22	x		x			
Participant 23	x		x			
Participant 24	x	x	x			
Participant 25	x		x			
Participant 26	x	x	x			
Participant 27	x	x	x			
Participant 28	x	x	x			
Participant 29	x		x			
Participant 30	x	x	x			
Participant 31	x		x			
Participant 32	x	x	x			
Participant 33	x		x			
Participant 34	x	x	x			
Participant 35	x	x	x			
